# Number of infection events per cell during HIV-1 cell-free infection

**DOI:** 10.1038/s41598-017-03954-9

**Published:** 2017-07-26

**Authors:** Yusuke Ito, Azaria Remion, Alexandra Tauzin, Keisuke Ejima, Shinji Nakaoka, Yoh Iwasa, Shingo Iwami, Fabrizio Mammano

**Affiliations:** 1 Department of Biology, Faculty of Sciences, Kyushu University, Fukuoka, 819-0395 Japan; 2 INSERM, U941, 75010 Paris, France; 3 Univ Paris Diderot, Sorbonne Paris Cité, IUH, 75010 Paris, France; 4 Institut Universitaire d’Hématologie, Hôpital Saint-Louis, 75010 Paris, France; 5 School of Public Health, University of Alabama at Birmingham, Alabama, 35294-0011 USA; 6 PRESTO, JST, Saitama, 332-0012 Japan; 7 Institute of Industrial Science, The University of Tokyo, Meguro-ku, Tokyo, 153-8505 Japan; 8 CREST, JST, Saitama, 332-0012 Japan

## Abstract

HIV-1 accumulates changes in its genome through both recombination and mutation during the course of infection. For recombination to occur, a single cell must be infected by two HIV strains. These coinfection events were experimentally demonstrated to occur more frequently than would be expected for independent infection events and do not follow a random distribution. Previous mathematical modeling approaches demonstrated that differences in target cell susceptibility can explain the non-randomness, both in the context of direct cell-to-cell transmission, and in the context of free virus transmission (Q. Dang *et al*., Proc. Natl. Acad. Sci. USA 101:632-7, 2004: K. M. Law *et al*., Cell reports 15:2711-83, 2016). Here, we build on these notions and provide a more detailed and extensive quantitative framework. We developed a novel mathematical model explicitly considering the heterogeneity of target cells and analysed datasets of cell-free HIV-1 single and double infection experiments in cell culture. Particularly, in contrast to the previous studies, we took into account the different susceptibility of the target cells as a continuous distribution. Interestingly, we showed that the number of infection events per cell during cell-free HIV-1 infection follows a negative-binomial distribution, and our model reproduces these datasets.

## Introduction

The Human Immunodeficiency Virus type-1 (HIV-1) population in an infected individual is characterized by high genetic diversity that allows rapid adaptation to the changing environment, such as the development of an immune response or the initiation of an antiretroviral therapy. Genetic recombination events participate in the continuous production of these viral variants. For recombination to take place, distinct viruses must infect the same cell, and then different genomes must be packaged into a single virion so that the reverse transcription process can generate a chimeric viral genome by template switching (reviewed in ref. 1). By mixing the viral genomes, in one step, recombination creates new variants whose adaptation to the environment may exceed those of the parental viruses^2, 3^. This process could participate in the unfavourable prognosis of patients infected by two strains of HIV, known as double infection^4^. Although the majority of HIV-infected lymphocytes in the peripheral blood of patients carry only one viral genome copy^5, 6^, the epidemiologic spread of circulating recombinant forms (CRF) of HIV-1 demonstrates that recombination, and thus double infections, take place in infected patients^7^. It was also shown that recombination in HIV-infected patients may rescue defective viral genomes that carry drug resistance mutations^8, 9^.

The frequency of cells carrying multiple viral genomes is influenced by the virus transmission route. HIV-1 infection can spread either by cell-free virus particles or by a cell-associated process, in which viral particles and cellular receptors converge at the donor- and target-cell contact sites^10, 11^. Previous work has established that cell-associated HIV-1 transmission leads to frequent multiple infection events, while the majority of cells infected by free virions carry a single genome^12^. The genomes transmitted by one infected cell via the cell-mediated pathway, however, are expected to be very similar, thus reducing the likelihood that recombination will produce chimeric variants with new properties following this transmission method. Interestingly, despite the difference in efficacy, both virus transmission pathways result in a higher frequency of double-infected cells than would be expected for independent transmission events, showing that these infections do not follow a random distribution^13–17^. Two previous studies proposed that differences in cell susceptibilities could justify the experimental observation of double infection frequencies that are higher than expected for independent infection events^13, 18^. In one study, the authors considered only either “susceptible” or “unsusceptible” cell populations, and using a mathematical model found that heterogeneity in target cell susceptibility could account for the observation that more double infections occur *in vivo* than predicted by random models^18^. In that report, the percentage of susceptible cells in a target cell population was estimated to be 2.76%. In the other study, the cell population was considered as composed of a discrete number of subpopulations characterized by distinct susceptibility levels, and for simplicity 5 subpopulations of the same size were considered^13^. Here, by considering susceptibility as a continuous variable, we expand on those original reports, and provide a more detailed quantitative framework. We describe a novel mathematical model that explicitly considers the heterogeneity of target cells as a continuous variable. By fitting the model to experimental datasets of cell-free HIV-1 single and double infections, we show that the number of infection events per cell follows a negative-binomial distribution. We also quantified the increase in the double and multiple infection events as a function of the amount of inoculated virus, and we found that a significant proportion of cells can be infected by multiple genomes following cell-free HIV-1 exposure. Together, our results re-evaluate the potential impact of cell-free HIV-1 infection on HIV-1 genetic recombination.

## Materials and Methods

### Cells and proviral plasmids

HEK293T cells were maintained in Dulbecco modified Eagle’s medium (DMEM) supplemented with 10% heat-inactivated foetal calf serum (FCS) and antibiotics (100 IU/ml penicillin and 100 μg/ml streptomycin). MT4R5 cells^19^ were grown in RPMI-1640 medium supplemented with 10% heat-inactivated FCS, 100 IU/ml of penicillin, 100 µg/ml of streptomycin, and 0.25 μg/ml of amphotericin B. All cultures were maintained at 37 °C in a humidified atmosphere with 5% CO_2_.

The proviral constructs used here were derived from previously published plasmids based on the pNL4-3 construct and each carried a sequence coding for either green fluorescent protein (GFP) or heat stable antigen (HSA) reporter proteins cloned before the *nef* gene, with an IRES sequence allowing concomitant expression of the viral and reporter proteins^20, 21^. To prevent virus spread in culture, we have modified these constructs by deleting 1.3 kb of the *env* gene (between the *Kpn*I and *Bgl*II sites^15^. To complement these proviral constructs, we used an HIV-1 Env-expresser plasmid in which HIV-1 (pNL4-3) Env, (as well as Tat and Rev) expression is under the control of a chimeric SRα promoter (SV40-early promoter and LTR from HTLV-I)^15^.

### Preparation of virus stocks, infection, and datasets

Stocks of viruses expressing either GFP or HSA were prepared by transfecting sub-confluent 293-T cells in T75 flasks by JetPei (Polyplus Inc. Illkrich, France), following the manufacturer’s instructions. Medium was changed 16 h later, and the virus-containing supernatant was collected 40 h post-transfection and overlaid on a 20% sucrose cushion in a Beckman SW32 tube, after which particles were pelleted by centrifugation (98,000 g, 4 °C) for 90 min. Viral pellets were re-suspended in RPMI medium with FCS to obtain a 10-fold concentration as compared with the initial amount present in the culture supernatant, separated into several aliquots, and frozen at −80 °C. One day before infection, 2.0 × 10^5^ MT4R5 cells per well were seeded in a 96-well plate. Cells were then exposed to two-fold dilutions of one virus (single infection) or of both viruses at the same time (coinfection), using the indicated combinations of each virus input amount. Two hours after infection, cells were washed to eliminate excess virus and cultured for a total of 48 h. The percentage of GFP-positive cells was measured after cell fixation with 2% para-formaldehyde (PFA). HSA expression on the surface of infected cells was detected using a rat anti-HSA PerpCy5.5 antibody (Pharmingen, Le Pont-de Claix, France) before fixation in PFA. Flow-cytometry data were acquired using a FACSCalibur instrument (Becton Dickinson, Le Pont-de Claix, France) with CellQuest software and were analysed using FlowJo software (Treestar, Ashland, OR, USA). Cell viability was measured in parallel and found to be stable for at least 72 h post-infection (data not shown).

### Calculation of the frequencies in different FACS quadrants

**Figure 1:**
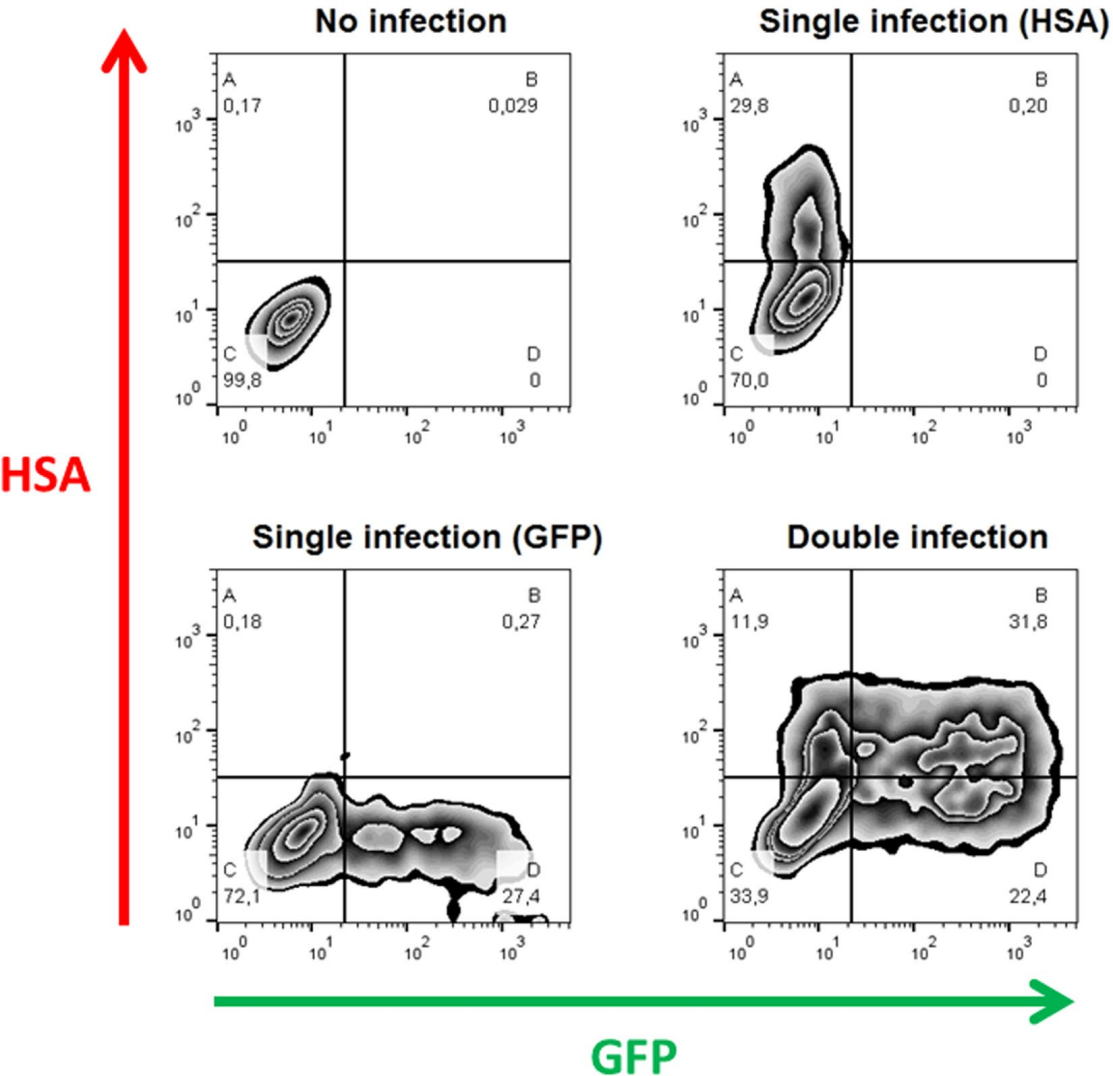
Flow cytometry analysis of single and double HIV-1 infection. Panels represent the following conditions, clockwise starting from the upper left panel: no infection; infection by the HSA virus; coinfection with HSA and GFP viruses, infection by the GFP virus. In each panel, the quadrants correspond to HSA^+^ (**A**); HSA^+^GFP^+^ (**B**); uninfected cells (**C**); and GFP^+^ (**D**). The percentage of cells in each quadrant is indicated under the letter identifying the quadrant.

Cells in a FACS graph can be divided into four quadrants, A, B, C, and D, based on their expression of GFP and/or HSA (Fig. 1). Cells in quadrant A express HSA only, those in quadrant B are positive both for HSA and GFP, cells in quadrant C are uninfected (i.e., negative both for HSA and GFP), and cells in quadrant D are positive for GFP only. Note that multiple infection events by viruses expressing the same reporter gene in quadrants A and D cannot be distinguished using FACS. Since for a given susceptibility parameter, *s*, the probability of a target cell being uninfected (i.e., no virus) is *e* ^−*βsV*^, the probability of a target cell being infected is 1−*e* ^−*βsV*^ ^16^. We assumed that the susceptibility parameter, *s*, obeys the Gamma distribution with the scale parameter, *p*, and the rate parameter, *q* (see Results for detailed calculations). Because two fluorescent proteins (i.e., HSA and GFP) are used, the term *V* is divided into ${\bar{V}}_{{\rm{HSA}}}$ and ${\bar{V}}_{{\rm{GFP}}}$, which represent the amount of effective HIV-1 expressing HSA and GFP, respectively. Additionally, to consider the case of each inoculated HIV-1 dataset (see Preparation of virus stocks and infection), we assumed ${\bar{V}}_{{\rm{HSA}}}={V}_{{\rm{HSA}}}$ and ${\bar{V}}_{{\rm{GFP}}}={V}_{{\rm{GFP}}}$ for 3.12 µl of inoculated HIV-1 expressing HSA and GFP, respectively, and ${\bar{V}}_{{\rm{HSA}}}={V}_{{\rm{HSA}}}\times r\ast$, and ${\bar{V}}_{{\rm{GFP}}}={V}_{{\rm{GFP}}}\times r\ast$, for the other amounts of inoculated HIV-1. We estimated the distribution of the following 11 parameters (*θ*): the shape parameter, *p*, the composed parameters, *βV*_HSA_/*q* and *βV*_GFP_/*q*, for single HSA and GFP HIV-1 experiments, respectively, and the scaling parameters r_*_ for 6.25, 12.5, 25, 37, 50, 75, 100, and 200 µl of the inoculated HIV-1 (Table 1) (i.e., $\theta =\{p,\beta {V}_{HSA}/q,\,\beta {V}_{GFP}/q,{r}_{6.25},{r}_{12.5},{r}_{25},{r}_{37},{r}_{50},{r}_{75},{r}_{100},{r}_{200}\}$). Therefore, under the assumptions, the theoretically predicted frequency of quadrant A, B, C, and D, respectively, in double HIV-1 infection experiments is calculated as follows:

$$\begin{array}{c}{F}_{A,co}(\theta )={\int }_{0}^{\infty }(1-{e}^{-\beta s{\bar{V}}_{{\rm{HSA}}}}){e}^{-\beta s{\bar{V}}_{{\rm{GFP}}}}\,\frac{{q}^{p}\,{s}^{p-1}}{{\rm{\Gamma }}(p)}{e}^{-qs}ds,\\ {F}_{B,co}(\theta )={\int }_{0}^{\infty }(1-{e}^{-\beta s{\bar{V}}_{{\rm{HSA}}}})(1-{e}^{-\beta s{\bar{V}}_{{\rm{GFP}}}})\frac{{q}^{p}\,{s}^{p-1}}{\Gamma (p)}{e}^{-qs}ds,\\ {F}_{C,co}(\theta )={\int }_{0}^{\infty }{e}^{-\beta s{\bar{V}}_{{\rm{HSA}}}}{e}^{-\beta s{\bar{V}}_{{\rm{GFP}}}}\frac{{q}^{p}\,{s}^{p-1}}{\Gamma (p)}{e}^{-qs}ds,\\ {F}_{D,co}(\theta )={\int }_{0}^{\infty }{e}^{-\beta s{\bar{V}}_{{\rm{HSA}}}}(1-{e}^{-\beta s{\bar{V}}_{{\rm{GFP}}}})\frac{{q}^{p}\,{s}^{p-1}}{\Gamma (p)}{e}^{-qs}ds.\end{array}$$

**Table 1:** Estimated parameters in the mathematical model of cell-free infection.

Parameters	Estimated values (mean)	95% CI
$p$	1.176	0.878–1.512
$\beta {\bar{V}}_{{\rm{HSA}}}/q$	$1.484\times {10}^{-2}$	$1.034\times {10}^{-2}$–$1.963\times {10}^{-2}$
$\beta {\bar{V}}_{{\rm{GFP}}}/q$	$2.115\times {10}^{-2}$	$1.486\times {10}^{-2}$–$2.796\times {10}^{-2}$
${r}_{6.25}$	$2.417$	$1.487$–$3.344$
${r}_{12.5}$	$4.231$	$2.588$–$5.812$
${r}_{25}$	$4.352$	$2.683\mbox{--}6.026$
${r}_{37}$	$11.09$	$7.100\mbox{--}15.16$
${r}_{50}$	$8.115$	$5.322\mbox{--}10.97$
${r}_{75}$	$12.79$	$8.485\mbox{--}17.42$
${r}_{100}$	$16.75$	$11.82\mbox{--}22.16$
${r}_{200}$	$26.87$	$17.49\mbox{--}36.81$

We further calculated and simplified those equations as follows:

1 $${F}_{A,co}(\theta )=\frac{1}{{(1+\frac{\beta }{q}{\bar{V}}_{{\rm{GFP}}})}^{p}}-\frac{1}{{(1+\frac{\beta }{q}({\bar{V}}_{{\rm{GFP}}}+{\bar{V}}_{{\rm{HSA}}}))}^{p}},$$

2 $${F}_{B,co}(\theta )=1-\,\frac{1}{{(1+\frac{\beta }{q}{\bar{V}}_{{\rm{HSA}}})}^{p}}-\,\frac{1}{{(1+\frac{\beta }{q}{\bar{V}}_{{\rm{GFP}}})}^{p}}+\frac{1}{{(1+\frac{\beta }{q}({\bar{V}}_{{\rm{HSA}}}+{\bar{V}}_{{\rm{GFP}}}))}^{p}},$$

3 $${F}_{C,co}(\theta )=\frac{1}{{(1+\frac{\beta }{q}({\bar{V}}_{{\rm{HSA}}}+{\bar{V}}_{{\rm{GFP}}}))}^{p}},$$

4 $${F}_{D,co}(\theta )=\frac{1}{{(1+\frac{\beta }{q}{\bar{V}}_{{\rm{HSA}}})}^{p}}-\frac{1}{{(1+\frac{\beta }{q}({\bar{V}}_{{\rm{HSA}}}+{\bar{V}}_{{\rm{GFP}}}))}^{p}}.$$

Notably, in the experiments with only HSA-expressing HIV-1 (i.e., single HSA HIV-1 experiments), we derived ${F}_{A,r}(\theta )=1-1/{(1+\beta {\bar{V}}_{{\rm{HSA}}}/q)}^{p}$, ${F}_{B,r}(\theta )=0$, ${F}_{C,r}(\theta )=1/{(1+\beta {\bar{V}}_{{\rm{HSA}}}/q)}^{p}$, and ${F}_{D,r}(\theta )=0$. Furthermore, in the single GFP HIV-1 experiments, we derived ${F}_{A,g}(\theta )=0$, ${F}_{B,g}(\theta )=0$, ${F}_{C,g}(\theta )=1/{(1+\beta {\bar{V}}_{{\rm{GFP}}}/q)}^{p}$, and ${F}_{D,g}(\theta )=$ $1-1/{(1+\beta {\bar{V}}_{{\rm{GFP}}}/q)}^{p}$ (see Data fitting, concerning the meaning of the index $r,g,co$).

### Data fitting

To fit the predicted frequency of each quadrant (i.e., Eqs (1–4)) with the experimental measurements by FACS analyses, we employed likelihood estimation to obtain an optimal set of parameter values. If we assume that the datasets used follow the Gaussian distribution with the mean ${\mu }_{i}$ and the variance ${\sigma }^{2}$ (i.e., $N({\mu }_{i},\,{\sigma }^{2}$)), the corresponding likelihood function is given as follows:

$$L(\rho ;x)=\prod \frac{1}{\sqrt{2\pi {\sigma }^{2}}}\,{e}^{-{\frac{(\mu -x)}{2{\sigma }^{2}}}^{2}}=\,\frac{1}{\sqrt{2\pi {\sigma }^{2}}}\,{e}^{-\frac{1}{2{\sigma }^{2}}{\sum }^{}{(\mu -x)}^{2}}\,.$$

Note that $\rho$ and $x$ represent the set of parameters and the measurements, respectively. The specific form of sum of squared residuals (SSR) is given by

$$\begin{array}{rcl}SSR(\theta ) & = & \sum _{r=1}^{10}\{{({F}_{A,r}-{\tilde{F}}_{A,r})}^{2}+{({F}_{C,r}-{\tilde{F}}_{C,r})}^{2}\}+\sum _{g=1}^{10}\{{({F}_{C,g}-{\tilde{F}}_{C,g})}^{2}+{({F}_{D,g}-{\tilde{F}}_{D,g})}^{2}\}\\  &  & +\sum _{co=1}^{18}\{{({F}_{A,co}-{\tilde{F}}_{A,co})}^{2}+{({F}_{B,co}-{\tilde{F}}_{B,co})}^{2}+{({F}_{C,co}-{\tilde{F}}_{C,co})}^{2}+{({F}_{D,co}-{\tilde{F}}_{D,co})}^{2}\}.\end{array}$$

Here, *θ* is the set of parameters needed to estimate. ${F}_{quadrant,r,g,co}\,\,$and ${\tilde{F}}_{quadrant,r,g,co}$ (*quadrant* = {A, B, C, D}) represent the predicted frequencies and the measurements by FACS analyses, respectively. The index *r*, *g*, *co* represent experiments with different amounts of inoculated HIV-1 (i.e., *r* = 1, …, 10 correspond to 3.12, 6.25, 12.5, 25, 37, 50, 75, 100, 100 and 200 µl, respectively, of single HSA HIV-1 experiments; *g* = 1, …, 10 correspond to 3.12, 6.25, 12.5, 25, 50, 50, 75, 100, 100 and 200 µl, respectively, of single GFP HIV-1 experiments; *co* = 1, …, 18 correspond to 25 and 25, 25 and 50, 50 and 25, 37 and 50, 50 and 50, 37 and 75, 75 and 50, 75 and 75, 25 and 100, 100 and 25, 37 and 100, 50 and 100, 100 and 50, 100 and 50, 75 and 100, 100 and 75, 100 and 100 µl and 100 and 100 µl, respectively, of double HSA and GFP HIV-1 experiments). If the variance ${\sigma }^{2}$ is constant, then likelihood estimation is completely determined by the SSR between theoretical values and measurements (i.e., ${\sum }^{}{(\mu -x)}^{2}$). However, to take into account for possible variations, we assume that the variance is not constant, and employed Bayesian inference approach with the Markov Chain Monte Carlo (MCMC) method to estimate distribution of parameters.

### Bayesian inference method for the parameter estimation

**Figure 2:**
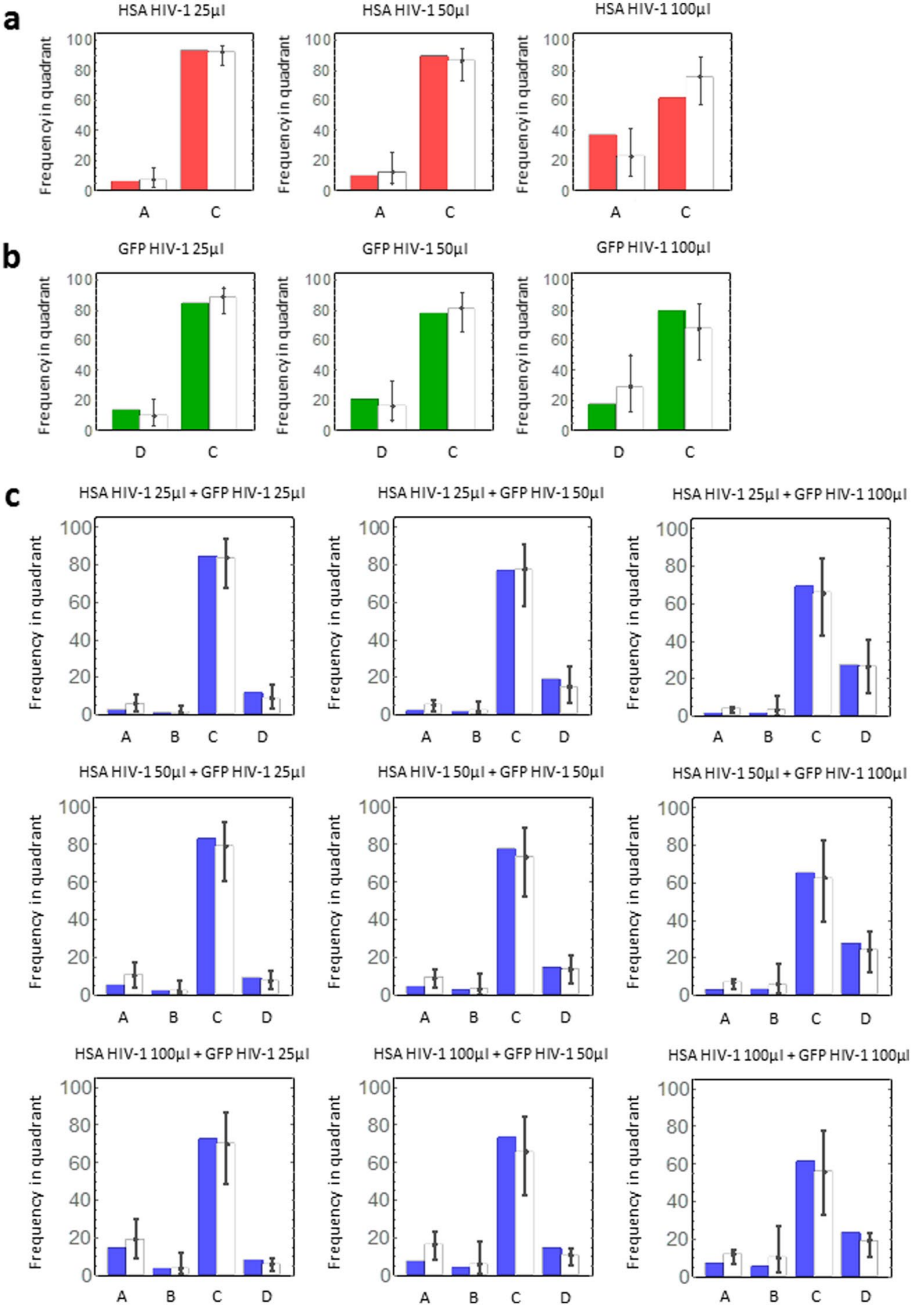
Frequency of single infection and coinfection: (**a)** The experimental and theoretical frequencies of quadrants A (i.e., HSA-positive) and C (i.e., HSA-negative) in three independent experiments using only HSA HIV-1 are shown by red and white bars, respectively. **(b)** The experimental and theoretical frequencies of quadrants D (i.e., GFP-positive) and C (i.e., GFP-negative) in single GFP HIV-1 experiments are shown by green and white bars, respectively. **(c)** The experimental and theoretical frequencies of quadrants A (i.e., HSA-positive), B (i.e., positive both for HSA and GFP), C (i.e., negative both for HSA and GFP), and D (i.e., GFP-positive) in double HIV-1 experiments are shown by blue and white bars, respectively. Two independent experiments were run with the nine indicated combinations of HSA and GFP HIV-1 corresponding to all possible combinations of the three different amounts of each virus used in single experiments. Note that each error bar represents the 95% credible interval obtained from Markov Chain Monte Carlo (MCMC) parameter inferences (Table 1).

The R package FME enables one to perform MCMC sampling by “delayed rejection and adaptive Metropolis algorithm”^22^. In the framework of the FME package to perform Bayesian inference, the error between observations and model predictions is assumed to follow Gaussian distribution with the mean 0 and the variance ${\sigma }^{2}$. Moreover, the reciprocal of the variance (i.e., $1/{\sigma }^{2}$) follows a Gamma distribution, while the prior distribution of all parameters is a Gaussian distribution^22^. In this study, 120,000 MCMC samples were generated, and the first 20,000 chains were discarded as burn-in samples. Convergence of the Markov chain was manually checked by the output of the ‘traceplot’ and the histogram of the posterior distribution. As shown in Table 1, the 95% CI (credible interval) represents the range from 2.5% to 97.5% in each estimated distribution, and the mean value represents the one of each posterior distribution, and also the estimated posterior distributions are obtained by using the same random seed. The fits of Eqs (1–4) to the experimental data with different amounts of inoculated HIV-1 are shown in Fig. 2 and Fig. S1. Our estimated parameter values are summarized in Table 1.

## Results

### The number of infection events during cell-free infection follows a negative-binomial distribution

It was previously demonstrated that cells infected by more than one virus occur at a frequency higher than that expected by Poisson distribution^12, 13, 15–17^. Here, we developed a novel mathematical model explicitly considering the heterogeneity of target cells susceptibility to infection. In our model, we define the following parameters: *V* is the amount of effective virus for infection events, *β* is the infection rate of HIV-1, and *s* is the susceptibility of the target cells to HIV-1 infection. The probability of a target cell being infected (i.e., carrying and expressing an integrated HIV genome) by *n* viruses can be determined by Poisson distribution as previously described^12, 16, 17^:

5 $$f(X=n\,;\,\beta sV)=\frac{{(\beta sV)}^{n}\,{e}^{-\beta sV}}{n!}.$$

Additionally, to consider the heterogeneity of target cell susceptibility^13, 15^, we assumed that the susceptibility parameter, $s$, obeys the following Gamma distribution^23^:

6 $$g(s;\,p,\,q)=\frac{{q}^{p}\,{s}^{p-1}{e}^{-sq}}{{\Gamma }(p)}.$$

It is well known that Gamma distribution can approximate any one-peak distribution and reproduce a variety of biological phenomena^23–25^. Here *p* > 0 and *q* > 0 are the shape and rate parameters, and *Γ*(*) is the gamma function. In a previous study^13^, it was artificially assumed that there are a finite number of subpopulations of cells with different susceptibilities to infection (i.e., five discrete susceptible populations). We extended that assumption to a continuous range of susceptible populations allowing our model to reflect, for example, the level of expression of CD4 and/or co-receptors on target cells, which are widely but continuously distributed^14, 26, 27^. Using Eqs (5, 6), we calculated the probability density function for a cell being infected by *n* HIV-1 in a heterogeneous target cell population:

7 $$\begin{array}{rcl}{\Pr }\,(X=n;p,1/(1+\frac{\beta V}{q})) & = & {\int }_{0}^{{\infty }}\frac{{(\beta sV)}^{n}{e}^{-(\beta sV)}}{n!}\frac{{q}^{p}{s}^{p-1}}{{\Gamma }(p)}{e}^{-qs}ds,\\  & = & {(\beta V)}^{n}{q}^{p}\frac{1}{n!{\Gamma }(p)}{\int }_{0}^{{\infty }}{s}^{n+p-1}\,{e}^{-(\beta V+q)s}\,ds,\\  & = & \frac{{\Gamma }(n+p)}{n!{\Gamma }(p)}{(\frac{q}{\beta V+q})}^{p}{(\frac{\beta V}{\beta V+q})}^{n},\,\\  & = & (\begin{array}{c}n+p-1\\ n\end{array}){(\frac{1}{1+\frac{\beta V}{q}})}^{p}{(\frac{\frac{\beta V}{q}}{1+\frac{\beta V}{q}})}^{n}.\end{array}$$

Therefore, the number of HIV-1 infection events per cell during cell-free HIV-1 infection (i.e., ${\Pr }(X=n)$) follows a negative-binomial distribution of mean $\beta pV/q$ and variance $\beta pV/q(1+\beta V/q)$.

We estimated the parameters in Eq. (7) by fitting Eqs (1–4) to the experimental measured frequencies of quadrants A, B, C, and D in our FACS analyses (see MATERIALS AND METHODS), and these values are summarized in Table 1. A set of representative analyses is shown in Fig. 2. In these panels, the coloured and white bars represent experimental measurements and theoretical predictions, respectively. In both single (Fig. 2a,b) and double HIV-1 infection experiments (Fig. 2c), our mathematical model reproduces all experimental datasets well. An independent set of data and the corresponding analysis are shown in Supplemental Figure 1, both for single and double infection experiments.

**Figure 3:**
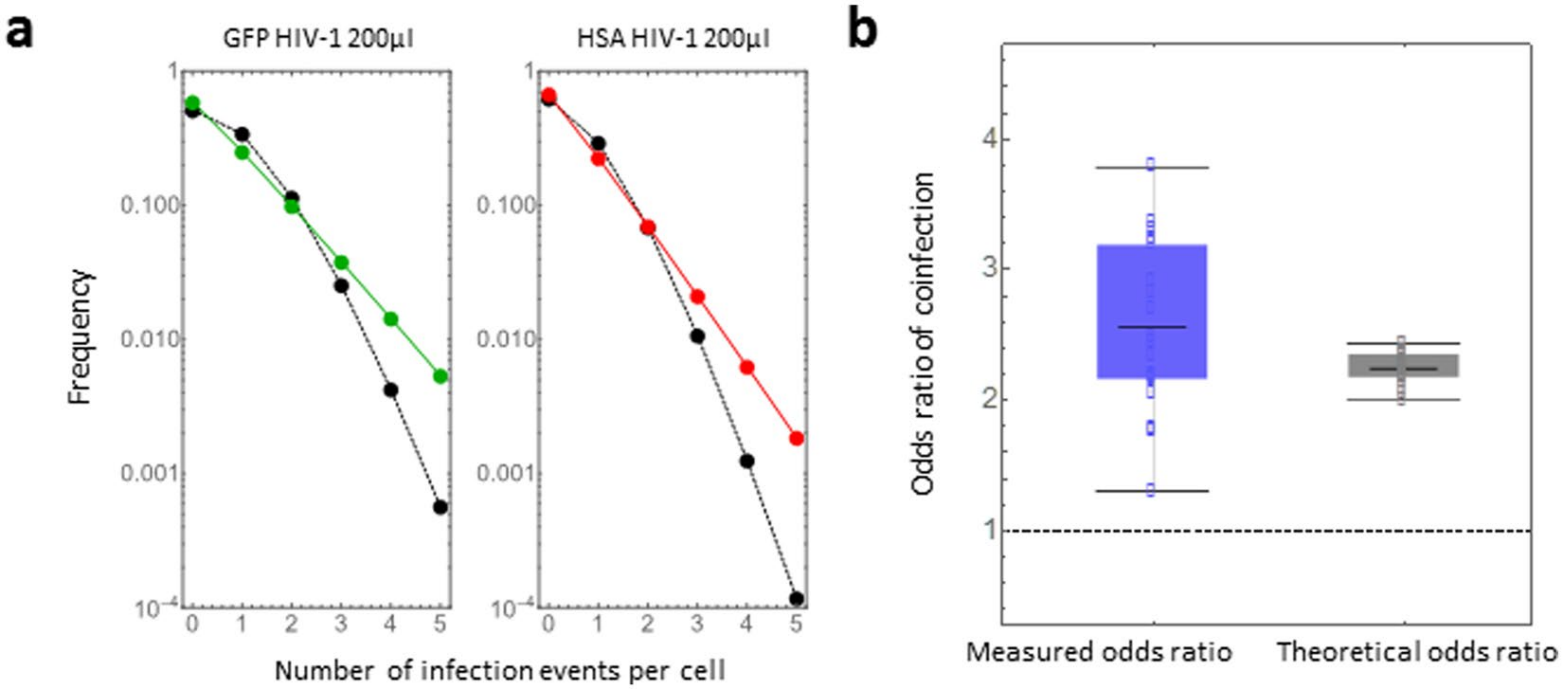
Frequency of multiple infection events per cell: (**a**) The expected negative-binomial distributions of the number of infection events per cell in 200 μl in GFP and HSA HIV-1 single experiments are shown in green and red curves, respectively. These curves were drawn using the mean derived from MCMC parameter inferences (Table 1). The black curves represent the expected Poisson distribution with the mean of the Gamma-distributed susceptibility parameter, $$s$$ (i.e., the target cell population is assumed to have homogeneous susceptibility). **(b)** The experimental odds ratio ($$O{R}_{E})$$ and theoretical odds ratio ($$O{R}_{M}$$, calculated by our estimated parameters) are shown in blue and black box plots, respectively. The dotted line corresponds to the odds ratio of 1 predicted by a Poisson distribution (i.e., $$O{R}_{M}=1$$: random HIV-1 infection).

The expected negative-binomial distributions of the number of infection events per cell in 200 μl for GFP and HSA HIV-1 single experiments are shown in green and red curves, respectively, as examples in Fig. 3a. The black curves represent the expected Poisson distribution with the mean of the Gamma distributed susceptibility parameter, *s* (i.e., the target cell population is assumed to have homogeneous susceptibility). While the mean and variance of a Poisson distribution are the same, the variance of a negative-binomial distribution is larger than its mean. This property of negative-binomial distribution explains that the more susceptible one cell is, the more effectively it will be infected by HIV-1^13, 14^.

### Calculation of the odds ratio in a cell-free HIV-1 infection

The frequency of co-infected cells with HIV-1 expressing HSA and GFP has previously been quantified by calculating the odds ratios^13–15^. The odds of HSA-positive cells being GFP-positive can be calculated by

$$[{\tilde{F}}_{B}/({\tilde{F}}_{A}+{\tilde{F}}_{B})]/\{1-[{\tilde{F}}_{B}/({\tilde{F}}_{A}+{\tilde{F}}_{B})]\}={\tilde{F}}_{B}/{\tilde{F}}_{A},$$

while the odds of HSA-negative cells being GFP-positive can be calculated by ${\tilde{F}}_{D}/{\tilde{F}}_{C}$. If the coinfection were random (i.e., independent events), then the ${\tilde{F}}_{B}/{\tilde{F}}_{A}$ and ${\tilde{F}}_{D}/{\tilde{F}}_{C}$. would be expected to be equal to 1, that is, the experimental odds ratio

$$O{R}_{E}=({\tilde{F}}_{B}/{\tilde{F}}_{A})/({\tilde{F}}_{D}/{\tilde{F}}_{C})={\tilde{F}}_{B}{\tilde{F}}_{C}/{\tilde{F}}_{A}{\tilde{F}}_{D}=1.$$

If coinfection occurred more or less frequently than that expected from random events, then the expected odds ratio would be $O{R}_{E} > 1$ and $O{R}_{E} < 1$, respectively. A higher frequency of coinfection has been experimentally confirmed in independent reports^13–15^. To study whether or not our novel model quantitatively reproduces this important property of HIV-1 coinfection, we derived the theoretical odds ratio, $O{R}_{M}$, from Eqs (1–4) as follows:

$$\begin{array}{rcl}O{R}_{M} & = & \frac{{F}_{B}({\boldsymbol{\theta }}){F}_{C}({\boldsymbol{\theta }})}{{F}_{A}({\boldsymbol{\theta }}){F}_{D}({\boldsymbol{\theta }})}\\  & = & 1+\frac{\frac{1}{{(1+\frac{\beta }{q}({\bar{V}}_{{\rm{HSA}}}+{\bar{V}}_{{\rm{GFP}}}))}^{p}}-\frac{1}{{(1+\frac{\beta }{q}{\bar{V}}_{{\rm{HSA}}})}^{p}{(1+\frac{\beta }{q}{\bar{V}}_{{\rm{GFP}}})}^{p}}}{\{\frac{1}{{(1+\frac{\beta }{q}{\bar{V}}_{{\rm{GFP}}})}^{p}}-\frac{1}{{(1+\frac{\beta }{q}({\bar{V}}_{{\rm{HSA}}}+{\bar{V}}_{{\rm{GFP}}}))}^{p}}\}\{\frac{1}{{(1+\frac{\beta }{q}{\bar{V}}_{{\rm{HSA}}})}^{p}}-\frac{1}{{(1+\frac{\beta }{q}({\bar{V}}_{{\rm{HSA}}}+{\bar{V}}_{{\rm{GFP}}}))}^{p}}\}}.\end{array}$$

The second term is always positive (see Supplementary Note), which implies that $O{R}_{M} > 1$ for all arbitrary parameter values. Therefore, under the condition of heterogeneous target cell susceptibility, our model always predicts that non-random HIV-1 coinfection occurs more frequently than would be expected for independent infection events (i.e., $O{R}_{M} > 1$). Notably, if the target cells have homogeneous susceptibilities (that is, the susceptibility parameter, $s$, is not distributed but fixed), then our model converges to a Poisson distribution and $O{R}_{M}=1$. In Fig. 3b, we compared the odds ratio measured by our experiments, $O{R}_{E}$, with the theoretical odds ratio, $O{R}_{M}$, which was calculated by our estimated parameters(Table 1). Thus, our novel model quantitatively reproduces the odds ratio and captures the known property of coinfection during cell-free HIV-1 infection *in vitro*.

### Quantification of infection events during cell-free HIV-1 infection

As discussed in previous work^12, 16^, some multiple infection events cannot be detected by FACS analyses because cells that are infected with one copy of HIV-1 expressing HSA and those carrying two copies of the same viral genome are similarly HSA-positive. Therefore, FACS analysis usually underestimates the true frequency of multiple infection^12^. As we derived ${F}_{quadrant,r,g,co}(r=1,\ldots ,10)(g=1,\ldots ,10)(co=1,\ldots ,18)$ in the MATERIALS AND METHODS, we calculated the number of infection events during cell-free HIV-1 infection, using Eqs (1–4) and our estimated parameters in Table 1. The frequency of cells infected by multiple HSA or GFP virions was calculated by the following equation, in which ${n}_{{\rm{HSA}}}$ and ${n}_{{\rm{GFP}}}$ are the number of infection events with HSA and GFP virions, respectively:

$${\int }_{0}^{\infty }\frac{{(\beta s{V}_{{\rm{HSA}}})}^{{n}_{{\rm{HSA}}}}\,{e}^{-\beta s{V}_{{\rm{HSA}}}}}{{n}_{{\rm{HSA}}}!}\frac{{(\beta s{V}_{{\rm{GFP}}})}^{{n}_{{\rm{GFP}}}}\,{e}^{-\beta s{V}_{{\rm{GFP}}}}}{{n}_{{\rm{GFP}}}!}\,\frac{{q}^{p}\,{s}^{p-1}}{\Gamma (p)}{e}^{-qs}ds.$$

**Figure 4:**
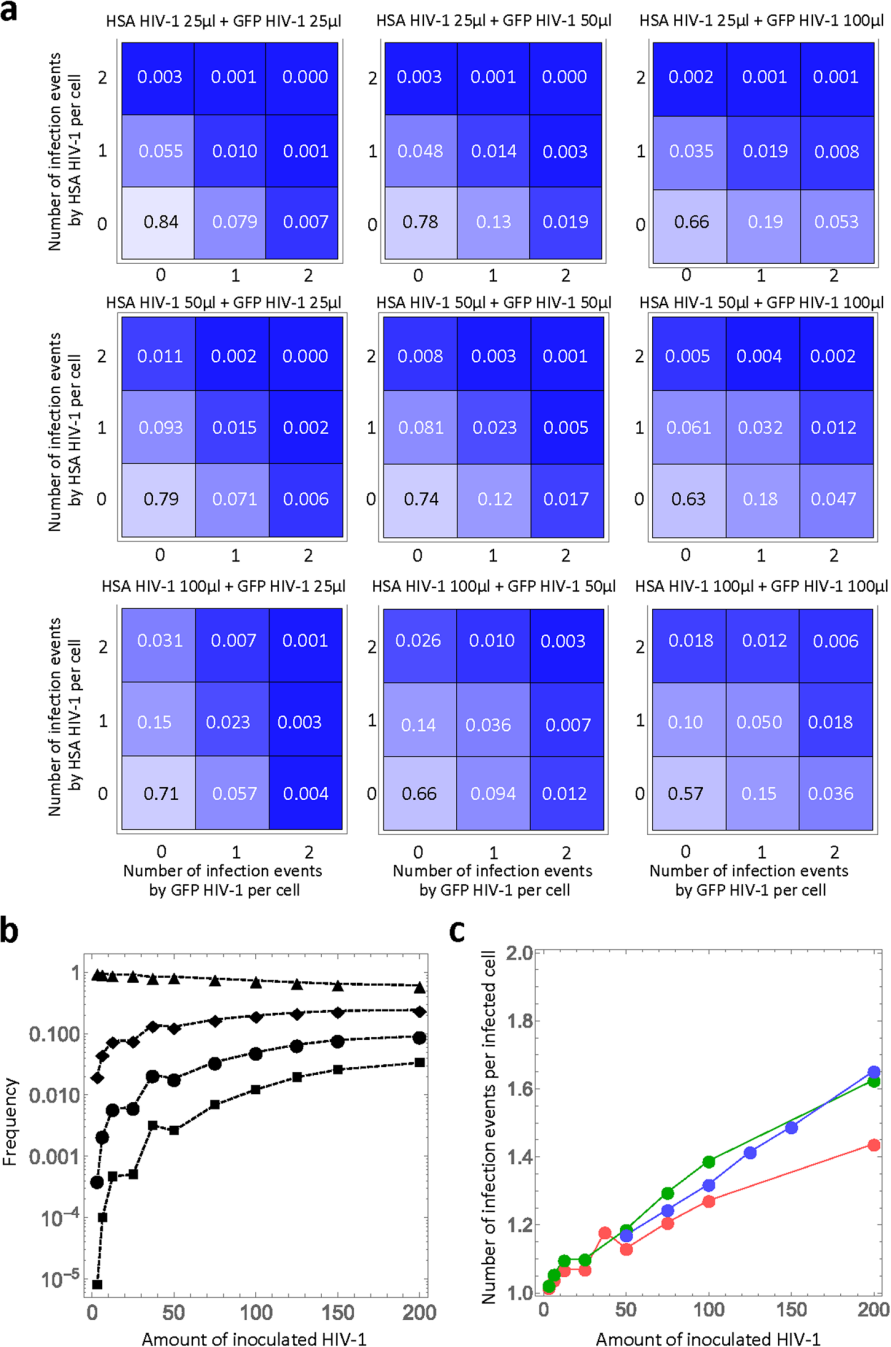
Quantitative analyses of multiple infection: (**a**) The distribution of the number of infection events per cell in double HIV-1 infection experiments with nine different combinations of virus amounts are shown. The number in each square is the estimated frequency of the corresponding infection events. **(b)** The mean frequency of the multiple infection events per cell inoculated with each different amount of HIV-1 is calculated. For double infection experiments, the amount is defined as the total inoculation of HSA and GFP HIV-1. The marks ▲, ◆, ●, and ■ show the mean frequencies of zero, one, two, and three infection events per cell, respectively. **(c)** The estimated mean number of infection events per infected cell with different amounts of HIV-1 is calculated. The red, green, and blue curves correspond to the experiments with HSA and GFP HIV-1 single infections, and double infections, respectively. In (**a**), (**b**), and (**c**), these calculations were all performed using the mean value obtained from MCMC parameter inferences (Table 1).

The estimated multiple infection frequencies in double HIV-1 infection experiments are shown in Fig. 4a (for other combinations of multiple infection, the frequency is less than 0.001). For example, in the experiment with 100 µl each of HSA and GFP HIV-1, although the experimentally measured frequency of coinfection was 5.47% (Fig. 2c, lower right panel, blue bar in **B**), our model revealed that 18.0% of the target cells were multiply infected (i.e., 1−(0.57 + 0.10 + 0.15)=0.18). Our estimated value during cell-free infection is smaller than the values previously estimated (21% in ref. 12) during cell-to-cell HIV-1 infection in cell culture. This analysis further supports that cell-to-cell infection enhances multiple infection events as compared with cell-free infection. In Fig. 4b, we calculated the mean frequency of multiple infection events following incubation with different amounts of HIV-1 in both single and double infection experiments. The marks ▲, ◆, ●, and ■ show the mean frequencies of zero, one, two, and three infection events per cell, respectively (data not shown for four or more infections events). As the amount of inoculated virus increases, the frequencies of multiple events consistently increase, and those of uninfected cells decrease. Interestingly, for these numbers of infection events, the frequencies reach steady state values around 150 µl of inoculated HIV-1. Thus, our quantitative analyses reveal the true frequency of multiple infection events and demonstrate that cell-free HIV-1 infection by itself induces multiple infection events. Furthermore, taking advantage of our modelling approach, we could estimate the mean number of infection events per infected cell during cell-free HIV-1 infection. In Fig. 4c, we found that the number increases from 1.02 to 1.65 as the amount of inoculated HIV-1 increases. Our estimated range is consistent with the previous observation of proviral copy number in infected cells measured by fluorescence *in situ* hybridization^12^.

## Discussion

In this study, we modelled the distribution of HIV infection events during cell-free infection *in vitro*, taking into account differences in susceptibility within the target cell population. Our model fits well with our collected experimental data for both single and double infections, indicating that the assumptions and the mathematical formulation successfully capture the biological processes underlying the distribution of HIV-1 infection events. More importantly, our mathematical model describes the hypothesis that variation in target cell susceptibility could account for non-random co-infection more accurately than previous work.

Two previous reports suggested that differences in cell susceptibilities could be responsible for the observed higher frequency of double infections, as compared to predictions based on the assumption of independent infection events^13, 18^. In those reports, cell susceptibility was either considered as a binary feature (cells were either susceptible or non-susceptible)^18^, or a limited number of subpopulations characterized by distinct susceptibility levels were considered^13^. Here, by considering susceptibility as a continuous variable, we expand on those original reports. The idea that the susceptibility of target cells is continuously distributed is intuitive because, even in cultured cells, it should be slightly different due to, for example, the change in the expression of (co-)receptors on each cell membrane over time. Employing this condition, we showed that the theoretical odds ratio (OR_M_) is always greater than 1 (see Fig. 3 and Supplementary Note). This result is consistent with the findings of previous work^13–15^. Hence, considering susceptibility as a continuous variable seems to be a more appropriate assumption for describing the cooperative nature of the HIV-1 infection process, for which several quantitative and qualitative parameters (number of receptors, availability of nucleotide pool, phase of the cell cycle, etc.) participate in defining the susceptibility of each cell within a population.

The approach described here may also be customized to describe other biological situations that display similar properties, for instance a distribution of eclipse period of virus-infected cells^24, 28^. Note that our model does not restrict the analysis to a situation in which the two events (infection by the GFP and the HSA virus, in our study) have the same efficiency. Indeed, for each virus we estimated a composed parameter ($\beta {\bar{V}}_{{\rm{HSA}}}/q$ or $\beta {\bar{V}}_{{\rm{GFP}}}/q$ in Table 1) to express the effective virus dose for a given volume of different viruses (e.g., 3.12 µl of inoculated HIV-1 expressing HSA or GFP). This choice increases the flexibility of our approach and allows an extension of its range of potential applications. Furthermore, we performed the experiments under conditions compatible with a large proportion of cells remaining uninfected to prevent saturation of the system and the potential associated biases. Of note, MT4 cells and their derivatives are highly susceptible to HIV infection. A gradual increase in the percentage of infected cells is observed when they are exposed to increasing virus doses, including conditions in which the majority of cells are infected. This feature allowed testing a wide range of experimental conditions, assisting the development of our model. Additionally, the model can be customized to work with less susceptible cells by experimentally determining the values of the parameters, $p$ and $q$, because these two parameters are strongly associated with the susceptibility in a target cell population.

Our model allows estimation of the frequency of single and multiple infection events for individual virus inputs. As shown in Fig. 3a (for 200 µl of each virus), our model predicts values that differ from the Poisson distribution; in particular, it predicts higher frequencies of multiple infection events. Also, our model predicts an $O{R}_{M}$ of coinfection that is always >1, reproducing the experimental observations from different groups^13–17^, while the Poisson distribution predicts an $O{R}_{M}$ = 1. Finally, having derived the values of the relevant parameters, our model allows the estimation of the frequency of multiple infections with the same virus, which are events that could not be experimentally determined in our system. Indeed, infection with one or more viruses carrying the same tag will produce similar distributions in the resulting FACS plots, preventing their experimental discrimination. In Fig. 4b, we quantified the frequency of multiple infection events for different amounts of inoculated HIV-1. As the inoculated amount increases, a significant proportion of cells can be infected by multiple viruses (e.g. ●/■). This demonstrates that cell-free HIV-1 infection by itself may have an important impact on driving the recombination of viruses. The range of infection events per infected cell predicted by our model in Fig. 4c fits the range of previously experimentally measured multiple infection events produced by cell-free HIV-1 infection using *in situ* hybridization^12^. Taken together, our findings and predictions lead to a more detailed understanding of the link between co-infection events and recombination.

An alternative mechanism was previously proposed to explain the disproportionate frequency of double-infected cells observed during co-infection experiments. The authors proposed that otherwise silent infection events were detected as a consequence of the additional Tat expression induced by the second virus^17^. We have previously demonstrated that in our experimental system this potential artefact did not play a role, since the use of lentiviral vectors that only expressed GFP resulted in similarly high frequencies of double-infected cells as those obtained using vectors that also encode Tat^15^.

In addition to these *in vitro* experimental and theoretical analyses, other mathematical models and computer simulations have been proposed to explain the observation of multiple infections *in vivo*. For instance, it was described that CD4^+^ T cells from the spleen of HIV-infected individuals carry on average 3.2 HIV proviral copies^29, 30^. Also, the number of multiply infected cells was found to correlate with the square of the overall number of infected cells^21, 31, 32^ in homogeneous target cell populations.

In agreement with previous reports^12^, we show here that multiple infection events take place with measurable frequencies following incubation with cell-free HIV-1 particles in a heterogeneous target cell population. Although the alternative pathway of infection that relies on cell contact-mediated infection is a more powerful transmission means, the impact of cell-free virus coinfection on HIV-1 genetic diversity may be expected to be more substantial because of the likelihood that spatially separated cells harbour genetically distinct variants. The impact on HIV-1 diversity, and consequently on the potential to adapt to a changing environment, are expected to correlate with the genetic distance between the parental variants. In the absence of antiretroviral treatment, the recurrent exposure of cells to infectious virions over the course of several years during this chronic infection creates numerous occasions for coinfections to happen. In view of these considerations, infection by cell-free virions emerges as a relevant means of HIV-1 diversification.

## Electronic supplementary material

Supplementary Information
