# Supplementary material for: Number of infection events per cell during HIV-1 cell-free infection
Source: Sci Rep. 2017 Jul 26;7:6559. doi: 10.1038/s41598-017-03954-9 (PMC5529392; doi:10.1038/s41598-017-03954-9)
Supplement: Supplementary file 1 — Supplementary Information [file 41598_2017_3954_MOESM1_ESM.pdf]

Supplementary Information

Number of infection events per cell during HIV-1 cell-free infection

Yusuke Ito[1,‡], Azaria Remion[2,3,4,‡], Alexandra Tauzin[2,3,4], Keisuke Ejima[5], Shinji Nakaoka[6,7], Yoh Iwasa[1], Shingo Iwami[1,6,7,†,*], & Fabrizio Mammano[2,3,4,†,*]

[1]Department of Biology, Faculty of Sciences, Kyushu University, Fukuoka 819-0395, Japan. [2]INSERM, U941, 75010 Paris, France. [3]Univ Paris Diderot, Sorbonne Paris Cité, IUH, 75010 Paris, France. [4]Institut Universitaire d'Hématologie, Hôpital Saint-Louis, 75010 Paris, France. [5]School of Public Health, University of Alabama at Birmingham, Alabama 35294-0011, USA. [6]PRESTO, JST, Saitama 332-0012, Japan. [7]Institute of Industrial Science, The University of Tokyo, Meguro-ku, Tokyo 153-8505, Japan. [8]CREST, JST, Saitama 332-0012, Japan.

‡,† These authors contributed equally to this study.

* Correspondence and requests for materials should be addressed to S.I. (email: siwami@kyushu-u.org) or F.M. (email: fabrizio.mammano@inserm.fr).

Supplementary Note: Calculation of theoretical odds ratio

We derived the theoretical odds ratio, OR_M, by Eqs. (1–4) as follows:

$$OR_M = \frac{F_B(\boldsymbol{\theta})F_C(\boldsymbol{\theta})}{F_A(\boldsymbol{\theta})F_D(\boldsymbol{\theta})}$$

$$= 1 + \frac{\dfrac{1}{\left(1+\dfrac{\beta}{q}(\bar{V}_{\mathrm{HSA}}+\bar{V}_{\mathrm{GFP}})\right)^p} - \dfrac{1}{\left(1+\dfrac{\beta}{q}\bar{V}_{\mathrm{HSA}}\right)^p\left(1+\dfrac{\beta}{q}\bar{V}_{\mathrm{GFP}}\right)^p}}{\left\{\dfrac{1}{\left(1+\dfrac{\beta}{q}\bar{V}_{\mathrm{GFP}}\right)^p} - \dfrac{1}{\left(1+\dfrac{\beta}{q}(\bar{V}_{\mathrm{HSA}}+\bar{V}_{\mathrm{GFP}})\right)^p}\right\}\left\{\dfrac{1}{\left(1+\dfrac{\beta}{q}\bar{V}_{\mathrm{HSA}}\right)^p} - \dfrac{1}{\left(1+\dfrac{\beta}{q}(\bar{V}_{\mathrm{HSA}}+\bar{V}_{\mathrm{GFP}})\right)^p}\right\}}.$$

Because $\beta > 0$, $p > 0$, $1/q > 0$, $\bar{V}_{\mathrm{HSA}} > 0$, $\bar{V}_{\mathrm{GFP}} > 0$ and

$$0 < 1+\frac{\beta}{q}(\bar{V}_{\mathrm{HSA}}+\bar{V}_{\mathrm{GFP}}) < \left(1+\frac{\beta}{q}\bar{V}_{\mathrm{HSA}}\right)\left(1+\frac{\beta}{q}\bar{V}_{\mathrm{GFP}}\right),$$

we obtained the following relationship of

$$0 < \left(1+\frac{\beta}{q}(\bar{V}_{\mathrm{HSA}}+\bar{V}_{\mathrm{GFP}})\right)^p < \left(1+\frac{\beta}{q}\bar{V}_{\mathrm{HSA}}\right)^p\left(1+\frac{\beta}{q}\bar{V}_{\mathrm{GFP}}\right)^p.$$

Thus, it is shown that

$$\frac{1}{\left(1+\dfrac{\beta}{q}(\bar{V}_{\mathrm{HSA}}+\bar{V}_{\mathrm{GFP}})\right)^p} - \frac{1}{\left(1+\dfrac{\beta}{q}\bar{V}_{\mathrm{HSA}}\right)^p\left(1+\dfrac{\beta}{q}\bar{V}_{\mathrm{GFP}}\right)^p} > 0\,.$$

Hence, the numerator in the second term of OR_M is always positive. Similarly, since

$$0 < 1+\frac{\beta}{q}\bar{V}_{\mathrm{GFP}} < 1+\frac{\beta}{q}(\bar{V}_{\mathrm{HSA}}+\bar{V}_{\mathrm{GFP}}),$$

we obtained

$$0 < \left(1+\frac{\beta}{q}\bar{V}_{\mathrm{GFP}}\right)^p < \left(1+\frac{\beta}{q}(\bar{V}_{\mathrm{HSA}}+\bar{V}_{\mathrm{GFP}})\right)^p,$$

this leads to

$$\frac{1}{\left(1+\dfrac{\beta}{q}\bar{V}_{\mathrm{GFP}}\right)^p} - \frac{1}{\left(1+\dfrac{\beta}{q}(\bar{V}_{\mathrm{HSA}}+\bar{V}_{\mathrm{GFP}})\right)^p} > 0.$$

This inequality explains why the denominator in the second term of OR_M is always positive. Therefore, we were able to show that $OR_M > 1$ holds.

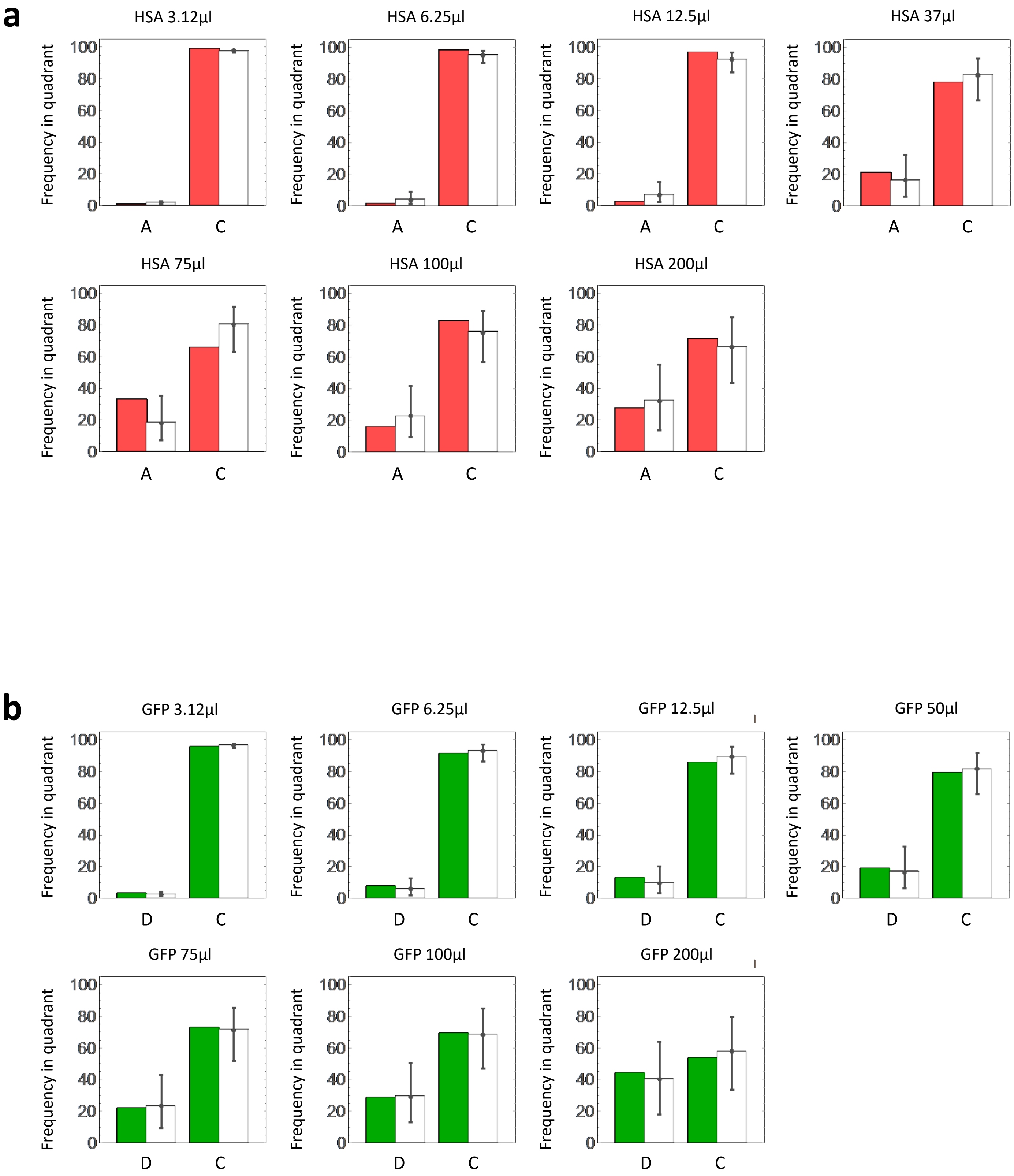

a
HSA 3.12µl
HSA 6.25µl
HSA 12.5µl
HSA 37µl
HSA 75µl
HSA 100µl
HSA 200µl
Frequency in quadrant
0
20
40
60
80
100
A
C
b
GFP 3.12µl
GFP 6.25µl
GFP 12.5µl
GFP 50µl
GFP 75µl
GFP 100µl
GFP 200µl
Frequency in quadrant
D
C

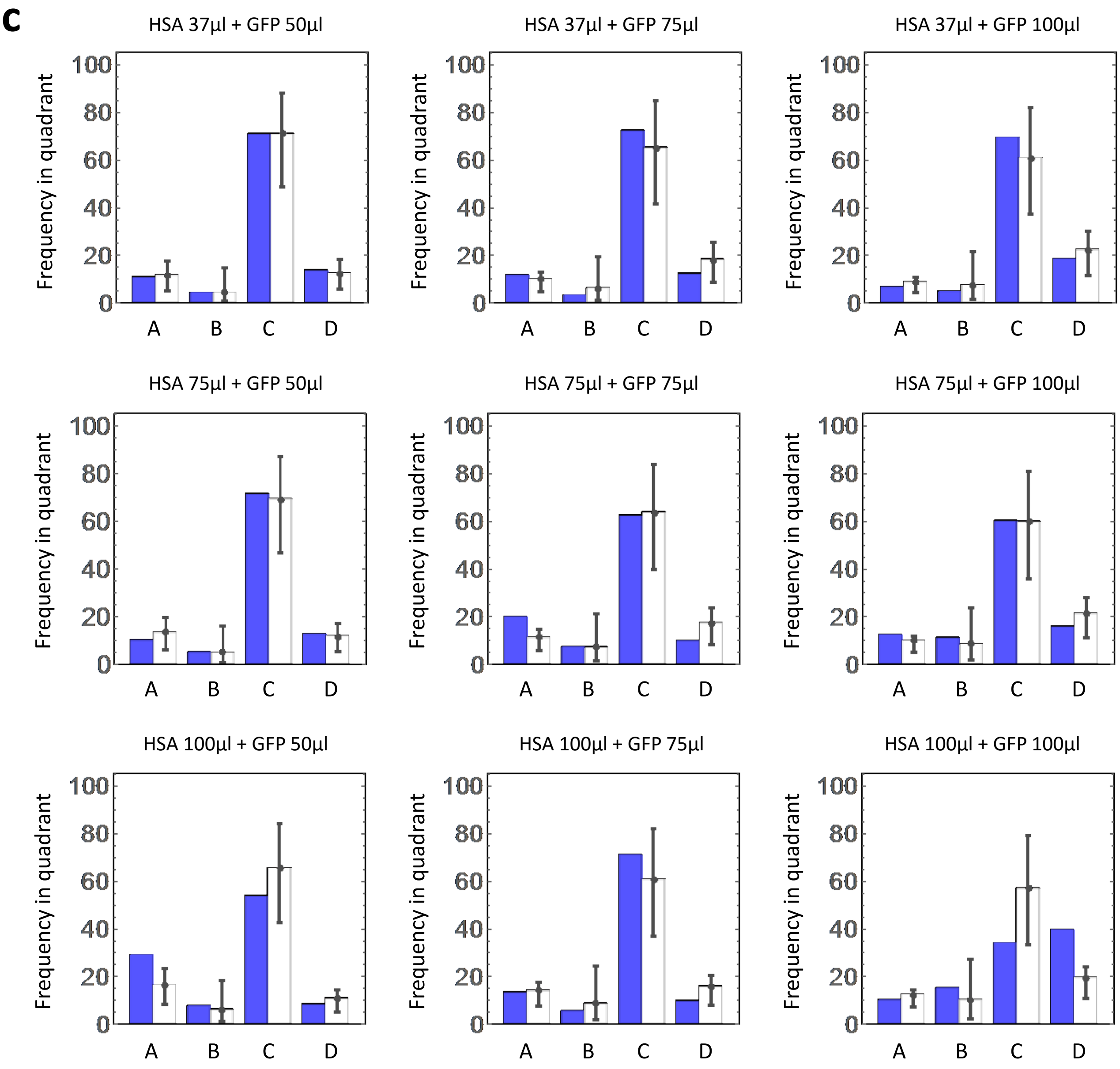

Figure S1 | Frequency of single infection and coinfection in other experiment: (a) The experimental and theoretical frequencies of quadrants A (i.e., HSA-positive) and C (i.e., HSA-negative) in seven independent experiments using only HSA HIV-1 are shown by red and white bars, respectively. **(b)** The experimental and theoretical frequencies of quadrants D (i.e., GFP-positive) and C (i.e., GFP-negative) in single GFP HIV-1 experiments are shown by green and white bars, respectively. **(c)** The experimental and theoretical frequencies of quadrants A, B (i.e., positive both for HSA and GFP), C (i.e., negative both for HSA and GFP), and D in double HIV-1 experiments are shown by blue and white bars, respectively, with different combinations of inoculated viral amount. Note that each error bar represents 95% credible interval obtained from Markov Chain Monte Carlo(MCMC) parameter inferences.

To examine how many HIV-1s infect T-cells in cell-free infection

Data

F_A : frequency of cells infected by HSA$^+$ HIV-1 only in quadrant A

F_B : frequency of cells infected by HSA$^+$ & GFP$^+$ HIV-1 in quadrant B

F_C : frequency of non-infected cells in quadrant C

F_D : frequency of cells infected by GFP$^+$ HIV-1 only in quadrant D

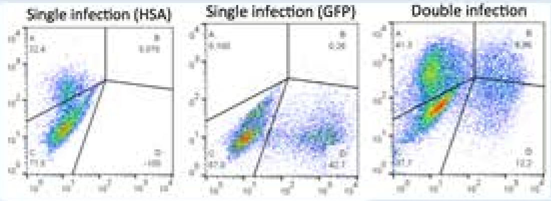

Details of the experiment

HSA	3.12µl	…	200µl	10 datasets
GFP	3.12µl	…	200µl	10 datasets
Double	25µl × 25µl	…	100µl × 100µl	18 datasets

Bayesian inference

Predicted value

$$F_A = \frac{1}{\left(1+\frac{\beta}{q}\bar{V}_{\mathrm{GFP}}\right)^p} - \frac{1}{\left(1+\frac{\beta}{q}(\bar{V}_{\mathrm{GFP}}+\bar{V}_{\mathrm{HSA}})\right)^p}$$

$$F_B = 1 - \frac{1}{\left(1+\frac{\beta}{q}\bar{V}_{\mathrm{HSA}}\right)^p} - \frac{1}{\left(1+\frac{\beta}{q}\bar{V}_{\mathrm{GFP}}\right)^p} + \frac{1}{\left(1+\frac{\beta}{q}(\bar{V}_{\mathrm{HSA}}+\bar{V}_{\mathrm{GFP}})\right)^p}$$

$$F_C = \frac{1}{\left(1+\frac{\beta}{q}(\bar{V}_{\mathrm{HSA}}+\bar{V}_{\mathrm{GFP}})\right)^p}$$

$$F_D = \frac{1}{\left(1+\frac{\beta}{q}\bar{V}_{\mathrm{HSA}}\right)^p} - \frac{1}{\left(1+\frac{\beta}{q}(\bar{V}_{\mathrm{HSA}}+\bar{V}_{\mathrm{GFP}})\right)^p}$$

Parameters

p $\quad r_{37}$

$\beta V_{HSA}/q$ $\quad r_{50}$

$\beta V_{GFP}/q$ $\quad r_{75}$

$r_{6.25}$ $\quad r_{100}$

$r_{12.5}$ $\quad r_{200}$

r_{25}

Unknown

Result

Distribution of the parameters

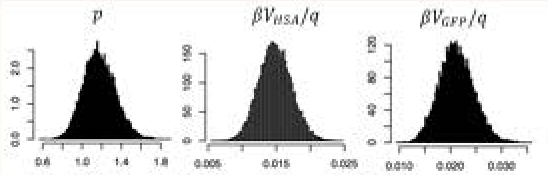

Prediction

Number of infection events

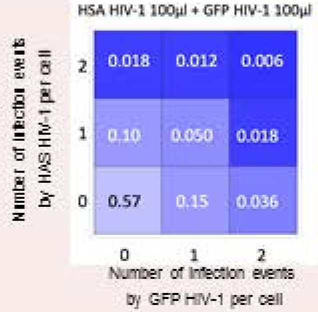

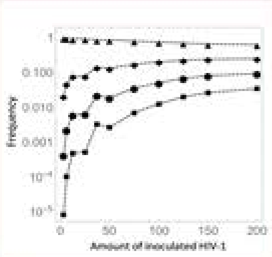

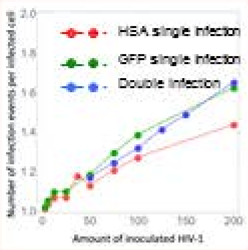

Figure S2 | Outline of our study strategy: (**Blue**) The frequency of cells in each quadrant were taken by FACS analyses in 3 independent experiments. (**Green**) We calculated the predicted frequencies corresponding to the measurements, and the 11 parameters are unknown. **(Red)** To estimate the distribution of the all parameters, the Bayesian method are applied. Using the estimated distribution, the calculations on the number of infection events were carried out.
